# Circulating MicroRNAs as Promising Diagnostic Biomarkers for Patients With Glioma: A Meta-Analysis

**DOI:** 10.3389/fneur.2020.610163

**Published:** 2021-02-01

**Authors:** Jimin He, Yao Jiang, Liang Liu, Zhihua Zuo, Chun Zeng

**Affiliations:** ^1^Department of Neurosurgery, Suining Central Hospital, Suining, China; ^2^Department of Clinical Laboratory Medicine, The Affiliated Hospital of Southwest Medical University, Luzhou, China; ^3^Department of Clinical Laboratory Medicine, Suining Central Hospital, Suining, China; ^4^Department of Neurosurgery, The Affiliated Hospital of Southwest Medical University, Luzhou, China

**Keywords:** microRNAs, glioma, diagnosis, biomarkers, meta-analysis

## Abstract

**Backgrounds and Purpose:** Currently, circulating microRNAs (miRNAs) are considered to be non-invasive diagnostic biomarkers in a broad range of tumors. Nevertheless, so far, miRNAs have not been fully applied to the clinic for routine screening in glioma patients. Thus, our goal is to evaluate the diagnostic performance of circulating miRNAs for gliomas via a meta-analysis. The present study is registered on the PROSPERO website, with the number CRD42020195883.

**Methods:** Literature retrieval was implemented in the PubMed, Embase, and Web of Science databases using the established search strategy. We pooled the sensitivity, specificity, and its 95% confidence intervals (CIs) for the included studies using the Stata 14.0 software. In addition, the heterogeneity between studies was assessed via the *Q* statistics and *I*^2^ values calculated by a Chi-square test. A bivariate random effects model was selected due to significant heterogeneity. Specifically, for exploring the factors influencing the heterogeneity, we implemented subgroup and meta-regression analyses. Ultimately, a Deek's funnel plot asymmetry test was used to estimate the potential publication bias.

**Results:** A total of 18 articles covering 24 studies were included, containing 2,170 glioma patients and 1,456 healthy participants. The overall pooled sensitivity, specificity, positive likelihood ratio (PLR), negative likelihood ratio (NLR), diagnostic odds ratio (DOR), and area under the curve (AUC) were 0.84 (95%CI: 0.79–0.87), 0.84 (95%CI: 0.80–0.88), 5.3 (95%CI: 4.1–6.8), 0.19 (95%CI: 0.15–0.25), 27 (95%CI: 18–41), and 0.91 (95%CI: 0.88–0.93), respectively. Additionally, the findings revealed that serum miRNAs and miRNA panels presented superior diagnostic performance.

**Conclusion:** Thus, circulating miRNAs have the potential to serve as diagnostic biomarkers for gliomas, but need to be verified via a large pool of prospective studies. Additionally, specific miRNAs still need to be elucidated in the diagnosis of a glioma, especially in the early screening stage. The findings may provide diagnostic and therapeutic strategies for the glioma population.

## Introduction

Gliomas originate from brain glial cells and are the most frequent type of intracranial primary tumors (1), accounting for 28% of all tumors (2), and 81% of malignant brain tumors (3). Based on a published statistical report of CBTRUS in 2019, the researchers found that the average annual age-adjusted incidence rate (AAAIR) of a glioma was approximately 7.87 per 100,000 from 2012 to 2016 in the United States (1). The astrocytoma and oligodendroglioma are the most commonly seen in the glioma population, on the basis of the World Health Organization (WHO) classification, gliomas are classified into a low-grade glioma (LGG, grades I-II), and a high-grade glioma (HGG, grades III-IV) (4, 5), and the WHO also incorporated molecular biomarkers for the sub-classification of gliomas in 2016 (6). According to previously established knowledge, an LGG had an approximate 43% possibility of 10-year survival (7). By contrast, once the patients suffering from an LGG progress to a HGG, especially glioblastoma (GBM) with grade IV, it has been reported that patients only have a 15-month average survival time (8). Thus, the early identification of gliomas is essential for clinicians.

Although computed tomography (CT) and magnetic resonance imaging (MRI) technologies (9) and comprehensive pathological diagnosis have emerged in the clinical setting, on account of exorbitant expenditure and invasive manipulation, these methods are not appropriate for routine screening in clinical practice. In recent years, multiple circulating biomarkers were employed to detect specific types of tumors earlier in the clinical setting, but these hematological molecules were not established in gliomas (10), where these blood-derived markers can function as minimally invasive biomarkers and support the diagnosis of tumors (11). In fact, clinically, there is also a lack of non-invasive, circulating, and feasible biomarkers for the diagnosis of gliomas (12). Thus, the construction of the circulating diagnostic biomarkers of gliomas is still the fundamental goal of improving its diagnosis and treatment.

At present, microRNAs (miRNAs), a category of small non-coding RNA, have aroused the unprecedented attention of the public. miRNAs, with a length of 19–25 nucleotides (nt) (13), combine with the 3'UTR of messenger RNAs (mRNAs) via complete complementary base-pairing, inhibiting the process of mRNA translation into protein, and also causing the degradation of mRNA via complete complementary base-pairing (14). It is of critical interest for miRNAs to regulate target genes, since they participate in various pathophysiological pathways and processes (13). Besides, miRNA expression alterations are the pathogenesis of most human malignancies, and the dysregulation of miRNAs also leads to tumor progression, in which miRNAs are regarded as oncogenes or tumor suppressors (15, 16). Similarly, some studies have confirmed that miRNAs played an imperative part in the tumorigenesis (17), progression, metastasis (18), and regulation of the cell cycle checkpoint (19), etc. Thus, many researchers proposed that miRNAs were capable of serving as non-invasive diagnostic biomarkers in a broad range of tumors, for instance, colorectal cancer (20, 21), osteosarcoma (22), breast cancer (23, 24), prostate cancer (25), cervical cancer (26), glioblastoma (27), and gliomas (28). Additionally, evidence from clinical research implied that dysregulated miRNAs were related to the development stage of gliomas (29). Most importantly, it has been demonstrated that miRNAs can be secreted into the blood or outside of cells (30), are stable in the blood circulation system, and present in a form of plasma or serum (31), as well as exosomes (32). Therefore, miRNAs may potentially act as diagnostic biomarkers for a glioma. However, some inconsistent conclusions make it challenging to propel the clinical use of miRNAs in the diagnosis of gliomas, which is pertinent to many categories of miRNAs, various analytic methods among those studies (33), as well as internal reference. Zhi et al. (34) revealed that a miRNA panel originating from serum for diagnosis of a glioma had sensitivity, specificity, and area under the curve (AUC) of 93.3%, 94.5%, and 0.9722 (95%CI: 0.9501–0.9942), respectively. Conversely, Xu et al. (35) found that plasma-derived miR-10b yielded only 44.6% sensitivity, 93.6% specificity, and 0.721 AUC (95%CI: 0.619–0.808). Similarly, Xiao et al. (36) also indicated the diagnostic efficacy of plasma miR-182 with a sensitivity of 58.5%, specificity of 85.2%, and AUC of 0.778 (95%CI: 0.679–0.878). Therefore, due to the present limitations in a single study, we conducted this meta-analysis, for the evaluation of the diagnostic value of circulating miRNAs in gliomas. If it is possible, we would like to investigate the diagnostic efficacy of specific miRNAs as glioma biomarkers for guiding clinical diagnosis.

## Materials and Methods

### Search Strategy

The PubMed, Embase, and Web of Science databases were searched by two reviewers (JH and YJ) to collect relevant articles with a deadline of August 18, 2020. Besides, there were no restrictions on the language and publication date. In regard to the literature retrieval strategy, Medical Subject Headings (MeSHs), including “glioma,” “microRNAs,” and “diagnosis” and their entry terms were combined for screening out all the original published studies. The search strategies are described in detail in Supplementary Table 1. Besides, literature listed on related reviews was also manually retrieved for fear that the important articles were omitted. Besides, when the key data were lacking, the two authors would contact the first author. The protocol of this present diagnostic meta-analysis have been registered on PROSPERO (CRD42020195883) and can be found at: https://www.crd.york.ac.uk/prospero/.

### Inclusion and Exclusion Criteria

Two reviewers (JH and YJ) independently performed the screening process through reading the titles and abstracts. Any disagreements were settled via discussion. According to the established inclusion and exclusion criteria, eligible articles were included based on the following criteria: (1) the studies were pertinent to miRNAs for the diagnosis of glioma patients; (2) there were complete data in each study used for calculating the value of true positives (TP), false positives (FP), false negatives (FN), and true negatives (TN); and (3) the sample size of each study was required to be at least 30. On the contrary, studies would be excluded when they were duplicates, reviews, meta-analyses, letters, abstracts, editorial, conference papers, studies without efficient data or irrelevant to the diagnosis of gliomas, as well as those with a sample size <30.

### Data Extraction

The general contents were extracted by two reviewers (JH and CZ), this information included the first author, country of participants, publication data (year), sample source, methods for detecting miRNAs expression, sample size, internal reference for relative quantitative analysis, truncation values, comparison type, and miRNA categories. In addition, the clinical and pathological characteristics of the glioma patients and healthy controls (HC) were also obtained, such as age, the proportion of male patients, WHO grade, tumor size, as well as Karnofsky performance scale (KPS). Most importantly, the information on sensitivity, specificity as well as sample size (including glioma patients and healthy subjects) was required for extraction in each study.

### Quality Assessment

Two authors (YJ and ZZ) separately implemented the quality assessment scale of the included 18 articles via employing the Quality Assessment of Diagnostic Accuracy Studies-2 (QUADAS-2) tool (37), which contains four domains: patient selection, index test, reference standard, and flow and timing. And every domain can be rated as a high (red), unclear (yellow), or low (green) risk bias. Similarly, applicability concerns in the first three domains were also evaluated through high, unclear, or low risk bias ratings (38).

### Statistical Analysis

The appraisal of the heterogeneity among studies was conducted via the *Q* statistics and *I*^2^ of Chi-square test, an *I*^2^ > 50% and *P* < 0.05 denoted the presence of heterogeneity, where an *I*^2^ ≤ 25%, 25% < *I*^2^ ≤ 50%, 50% < *I*^2^ ≤ 75%, and *I*^2^ > 75% was representative of modest, moderate, significant, and considerable heterogeneity, respectively (39, 40), if ever, the bivariate random-effects model was fitted for estimating the summary receiver operating characteristic (SROC) curve (41). This model integrates the inexplainable variability into the meta-analysis, takes the negative correlation between sensitivity and specificity into consideration (42), and meets the bivariate normal distribution via the logit transformation of sensitivity and specificity (43). The threshold effect was identified by a Spearman correlation coefficient and *P*-value (44), and a *P* < 0.05 was reflective of the existence of a threshold effect. Additionally, we divided all patients into seven subgroups, including sample source, dysregulated miRNAs, internal reference for relative quantitative analysis, comparison type, miRNA profiling, sample size, and cut-off values setting groups for subgroup and meta-regression analyses. Also, sensitivity analysis was implemented via four plots, which contained a quantile diagram of goodness-of fit on the basis of residual, a Chi-square probability graph of Mahalanobis distance squared, used to evaluate the bivariate normality hypothesis, a spike plot for screening out the studies with the most influencing overall diagnostic efficacy via Cook's distance method, and a scatter diagram for identifying the outliers (45). Additionally, we applied the Deek's funnel plot asymmetry test to explore whether publication bias existed, with *P* < 0.10 indicating the existence of publication bias, which is a key problem of diagnostic meta-analysis (46).

## Results

### The Characteristics of Studies

**Figure 1:**
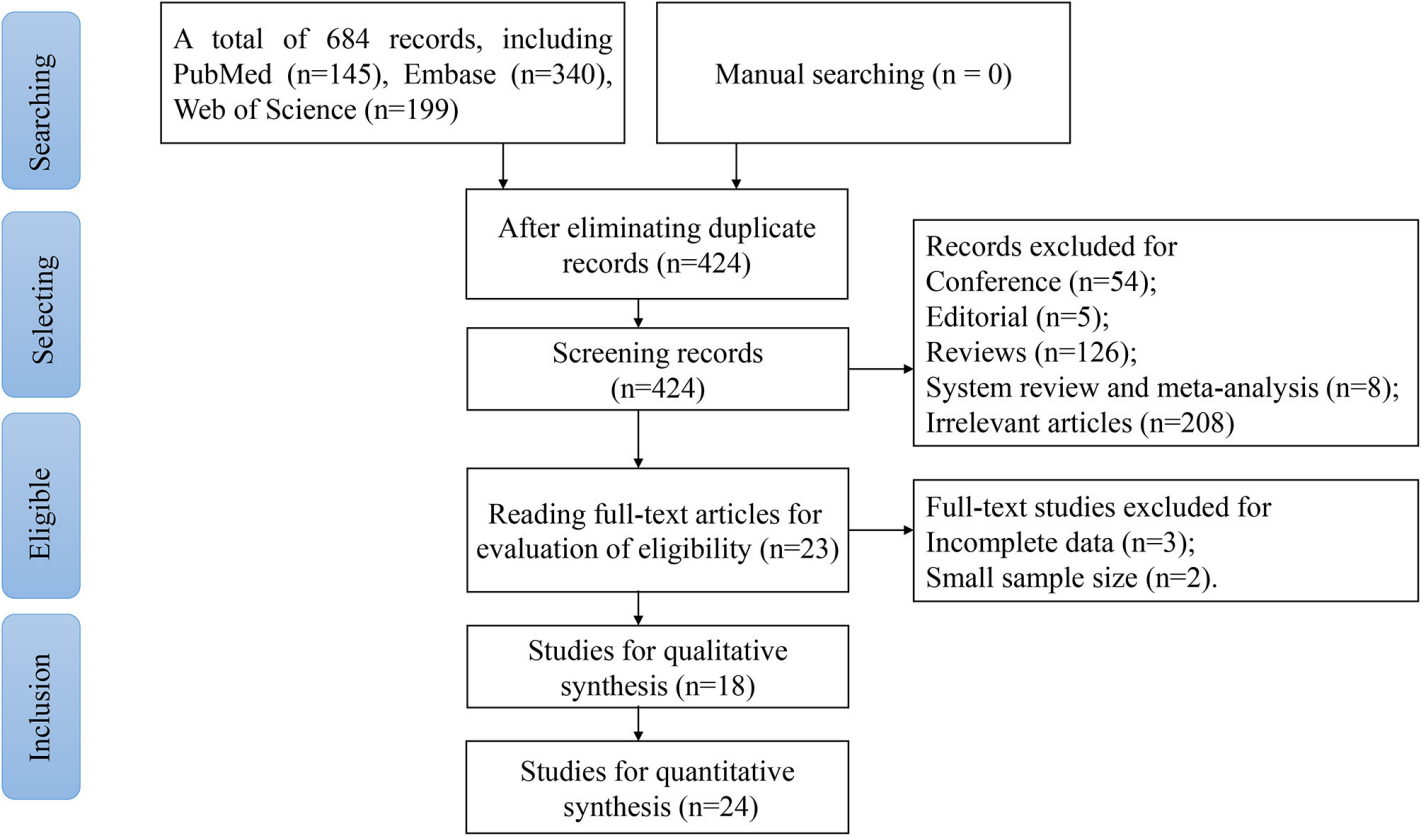
The flow chart of the study screening.

We found 684 studies in the PubMed, Embase, and Web of Science databases, then removed 260 duplicates. Through reading titles and abstracts, 401 studies were eliminated. Ultimately, after excluding five studies for incomplete data (*n* = 3) or a small sample size (*n* = 2), 18 eligible articles (28, 34–36, 47–60) containing 24 studies were employed for the meta-analysis. We depicted the flow diagram of the study screening according to the Preferred Reporting Items for Systematic Reviews and Meta-Analyses (PRISMA) (http://www.prisma-statement.org/) (Figure 1). Accordingly, 24 studies contained 2,170 glioma patients and 1,456 healthy participants in the present study. The main characteristics of each study and their diagnostic performance are described in Table 1. Furthermore, the clinical and pathological traits of participants in the included studies are shown in Table 2. The information of HC's age and the proportion of men in most of the studies was not given, where healthy individuals were described using age and gender-matched healthy volunteers.

**Table 1:** The characteristics of the 18 included articles.

**References**	**Ethnicity**	**Comparison type**	**Sample source**	**Dysregulated miRNAs**	**Method**	**Reference**	**miRNA profiling**	**Cut-off**	**Sample size**		**Sen**	**Spe**	**AUC (95%CI)**
									**Gliomas**	**HC**			
Chen et al. (47)	Asian	Glioma vs. HC	Plasma	Up	qRT-PCR	U6	miR-720	3.19	122	60	0.713	0.833	0.773 (0.706–0.832)
Huang et al. (48)	Asian	Glioma vs. HC	Serum	Down	qRT-PCR	U6	miR-376a	1.95	100	50	0.81	0.82	0.872
Huang et al. (48)	Asian	Glioma vs. HC	Serum	Down	qRT-PCR	U6	miR-376b	2.07	100	50	0.82	0.78	0.89
Huang et al. (48)	Asian	Glioma vs. HC	Serum	Down	qRT-PCR	U6	miR-376c	2.12	100	50	0.9	0.7	0.837
Lai et al. (49)	Asian	Glioma vs. HC	Serum	Up	qRT-PCR	miR-16-1	miR-210	2.259	126	40	0.9127	0.725	0.927 (0.889–0.964)
Lan et al. (28)	Asian	Glioma vs. HC	Serum	Up	qRT-PCR	miR-16	miR-301a	NA	60	43	0.862	0.932	0.937 (0.855–0.987)
Qi and Gao (50)	Asian	Glioma vs. HC	Serum	Down	qRT-PCR	U6	miR-33b	0.069	128	62	0.867	0.855	0.883
Shao et al. (51)	Asian	Glioma vs. HC	Plasma	Up	qRT-PCR	NA	miR-454-3p	NA	70	70	0.9905	0.8286	0.9063 (0.8487–0.9639)
Sun et al. (52)	Asian	Glioma vs. HC	Serum	Down	qRT-PCR	cel-miR-39	miR-128	7.139	151	53	0.8675	0.8868	0.9095 (0.8695–0.9496)
Tang et al. (53)	Asian	Glioma vs. HC	Plasma	Down	qRT-PCR	U6	miR-122	1.225	74	74	0.919	0.811	0.939
Wang et al. (54)	Asian	Glioma vs. HC	Serum	Up	qRT-PCR	U6/RNU48	miR-214	NA	100	100	0.9	0.71	0.885
Wang et al. (54)	Asian	HGG vs. HC	Serum	Up	qRT-PCR	U6/RNU48	miR-214	NA	62	100	0.7258	0.95	0.909
Wei et al. (55)	Asian	Glioma vs. HC	Serum	Down	qRT-PCR	miR-24	miR-125b	NA	33	33	0.7879	0.7576	0.839 (0.743–0.935)
Xiao et al. (36)	Asian	Glioma vs. HC	Plasma	Up	RT-qPCR	RNU6B	miR-182	1.56	112	54	0.585	0.852	0.778 (0.679–0.878)
Xu et al. (35)	Asian	Glioma vs. HC	Plasma	Up	qRT-PCR	U6	miR-17	3.03	47	45	0.893	0.553	0.787 (0.690–0.865)
Xu et al. (35)	Asian	Glioma vs. HC	Plasma	Up	qRT-PCR	U6	miR-130a	2.68	47	45	0.702	0.652	0.720 (0.617–0.807)
Xu et al. (35)	Asian	Glioma vs. HC	Plasma	Up	qRT-PCR	U6	miR-10b	10.1	47	45	0.446	0.936	0.721 (0.619–0.808)
Xu et al. (35)	Asian	Glioma vs. HC	Plasma	Up	qRT-PCR	U6	miR-panel	5.43	47	45	0.723	0.851	0.872 (0.787–0.932)
Yang et al. (56)	Asian	Glioma vs. HC	Serum	Down	RT-qPCR	U6snRNA	miRNA-panel	5.6085	133	80	0.88	0.9787	0.972 (0.954–0.990)
Yue et al. (57)	Asian	Glioma vs. HC	Serum	Down	qRT-PCR	has-miR-16	miR-205	0.16	64	45	0.863	0.922	0.935
Zhang et al. (58)	Asian	HGG vs. HC	Serum	Down	qRT-PCR	cel-miR-39	miR-145-5p	NA	117	50	0.846	0.78	0.895
Zhao et al. (59)	Asian	Glioma vs. HC	Serum	Down	qRT-PCR	RUN44	miR-451a	11.977	118	84	0.814	0.797	0.816
Zhi et al. (34)	Asian	Astrocytoma vs. HC	Serum	Up	qRT-PCR	NA	miR-panel	5.649	90	110	0.933	0.945	0.9722 (0.9501–0.9942)
Zhu et al. (60)	Asian	Glioma vs. HC	Serum	Up	RT-qPCR	U6	miR-193b	1.155	122	68	0.795	0.868	0.903

*HC, healthy controls; HGG, high grade glioma; up, upregulated; down, downregulated; NA, not available; Sen, sensitivity; Spe, specificity; AUC, area under the curve; CI, confidence interval*.

**Table 2:** The clinical and pathological characteristics of glioma cases and healthy controls in the included studies.

**References**	**Age (mean** **±** **SD)**		**Male (%)**		**WHO grade**				**Tumor size**		**KPS**	
	**Gliomas**	**HC**	**Gliomas**	**HC**	**I**	**II**	**III**	**IV**	**≤5 cm**	**≥5 cm**	**≤90**	**≥90**
Chen et al. (47)	48.6 ± 6.01	NA	63.93	NA	19	21	35	47	76	46	50	72
Huang et al. (48)	NA	NA	70.00	NA	10	20	30	40	68	32	40	60
Lai et al. (49)	44 (15–73)^*^	NA	55.56	NA	13	35	46	32	67	59	53	73
Lan et al. (28)	46 (30–72)^*^	NA	45.00	NA	10	11	12	27	NA	NA	NA	NA
Qi and Gao (50)	NA	NA	57.03	NA	69^a^		59^b^		58^c^	70^d^	48	80
Shao et al. (51)	47.2 ±5.6	48.4 ± 3.0	51.43	50.00	8	15	25	22	NA	NA	NA	NA
Sun et al. (52)	NA	NA	55.63	NA	24	23	43	61	NA	NA	NA	NA
Tang et al. (53)	NA	NA	52.70	NA	14	17	20	23	41	33	NA	NA
Wang et al. (54)	NA	NA	83.00	NA	38^a^		62^b^		66^e^	34^f^	49^g^	51^h^
Wei et al. (55)	NA	43.7 (31.3–55.1)^*^	60.61	60.61	11	11	11^b^		NA	NA	NA	NA
Xiao et al. (36)	NA	NA	64.29	NA	18	23	32	39	66	46	42^i^	70^j^
Xu et al. (35)	49.2 (27–74)^*^	45.4 (20–68)^*^	61.70	55.56	16^a^		31^b^		NA	NA	25^g^	22^h^
Yang et al. (56)	NA	46.8 ±11.5	58.65	63.75	15^†^	55	45	33	NA	NA	NA	NA
Yue et al. (57)	45 (30–72)^*^	45.2 ± 10.3	50.00	53.33	7	9	21	27	NA	NA	NA	NA
Zhang et al. (58)	NA	NA	72.65	NA	–		–	117	NA	NA	24^g^	93^h^
Zhao et al. (59)	NA	NA	60.17	NA	27	33	33	25	53	65	58	60
Zhi et al. (34)	NA	NA	52.22	51.82	–	28	38	24	NA	NA	NA	NA
Zhu et al. (60)	NA	NA	63.11	NA	59^a^		63^b^		68^c^	54^d^	83	39

a *Grades I+II*;

b *Grades III+IV*;

c *Tumor size ≤ 3 cm*;

d *Tumor size ≥ 3 cm*;

e *Tumor volume < 38.2 cm^3^*;

f *Tumor volume ≥ 38.2 cm^3^*;

g *KPS ≤ 70*;

h *KPS ≥ 70*;

i *KPS ≤ 80*;

j *KPS ≥ 80*;

* *Median (range)*;

† *Not use*.

*HC, healthy controls; KPS, Karnofsky performance scale; WHO, World Health Organization; NA, not available*.

### Quality Assessment

**Figure 2:**
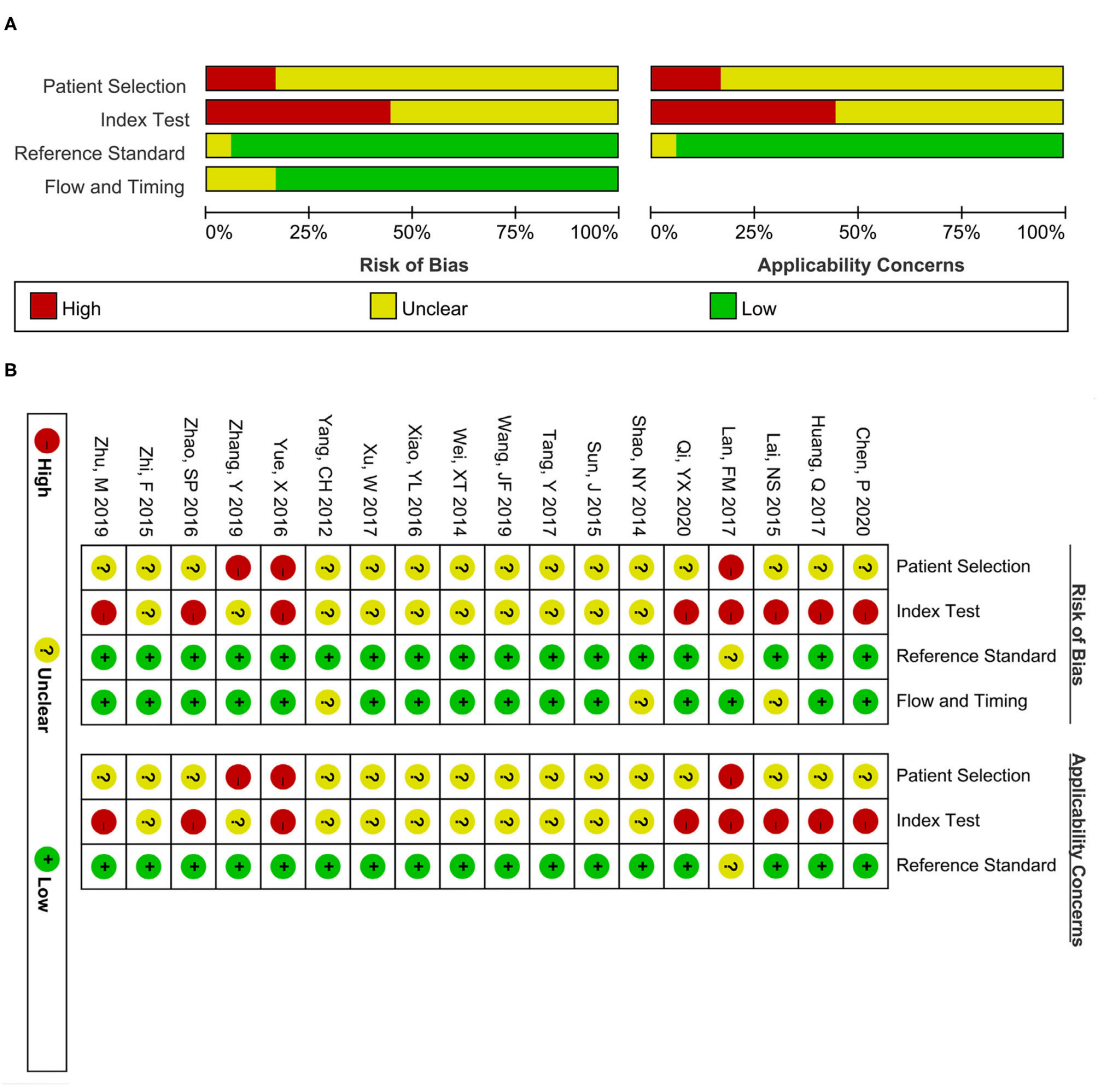
Quality assessment scale of the included studies. **(A)** Risk bias and applicability concerns graph. **(B)** Risk bias and applicability concerns summary. The red, yellow, and green colors separately present high, unclear, and low risk.

Figure 2 presents the risk evaluation for bias and clinical concerns. The domains of reference standard and time and flow were not influenced by risk bias. Given that all glioma patients in the included studies were pathologically confirmed, belonging to a case-control study design, most studies were rated as high risk or unclear risk in the first two domains.

### Diagnostic Accuracy of miRNAs in Gliomas

**Figure 3:**
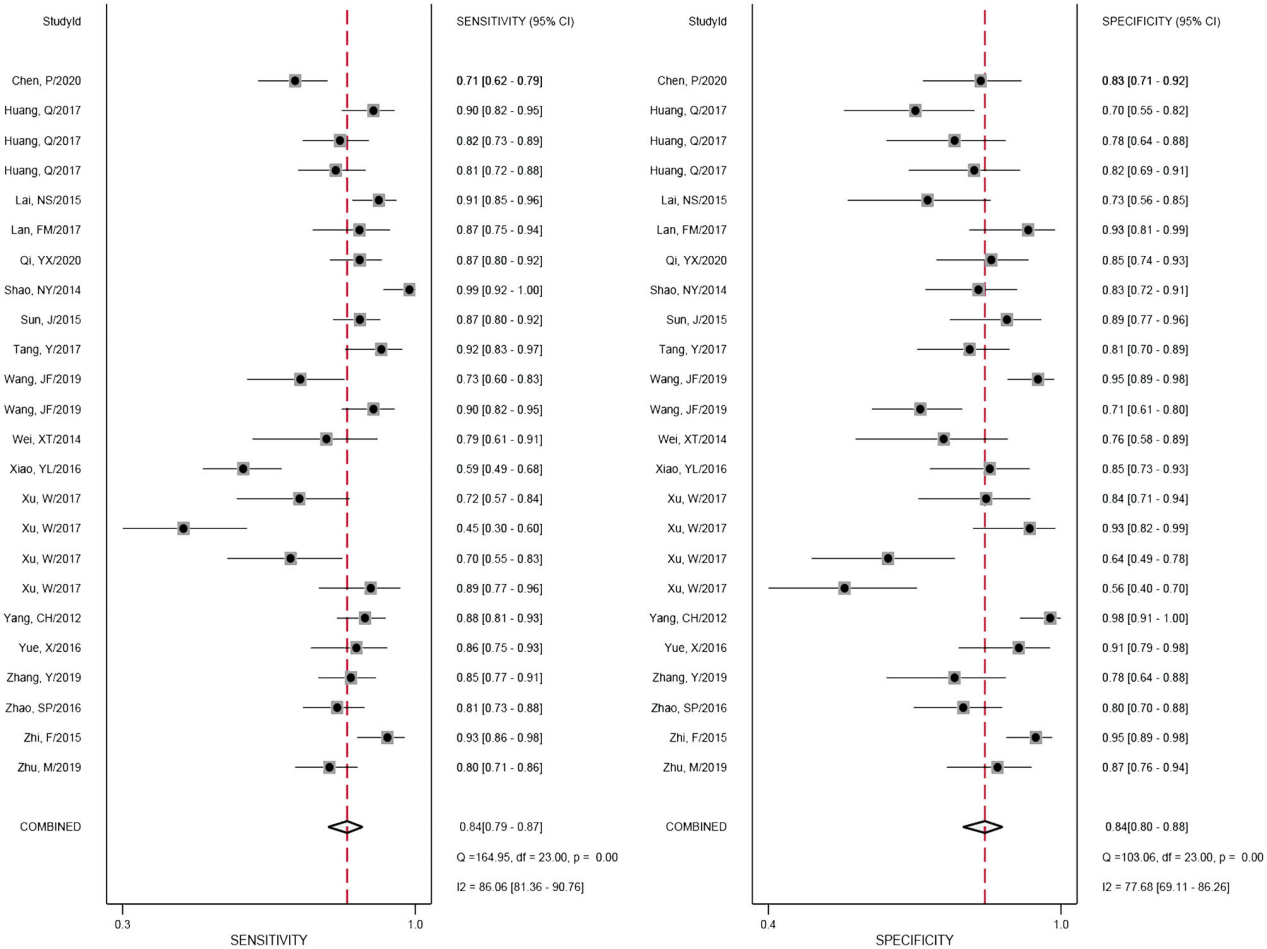
Forest plot of miRNAs for the diagnosis of gliomas.

**Figure 4:**
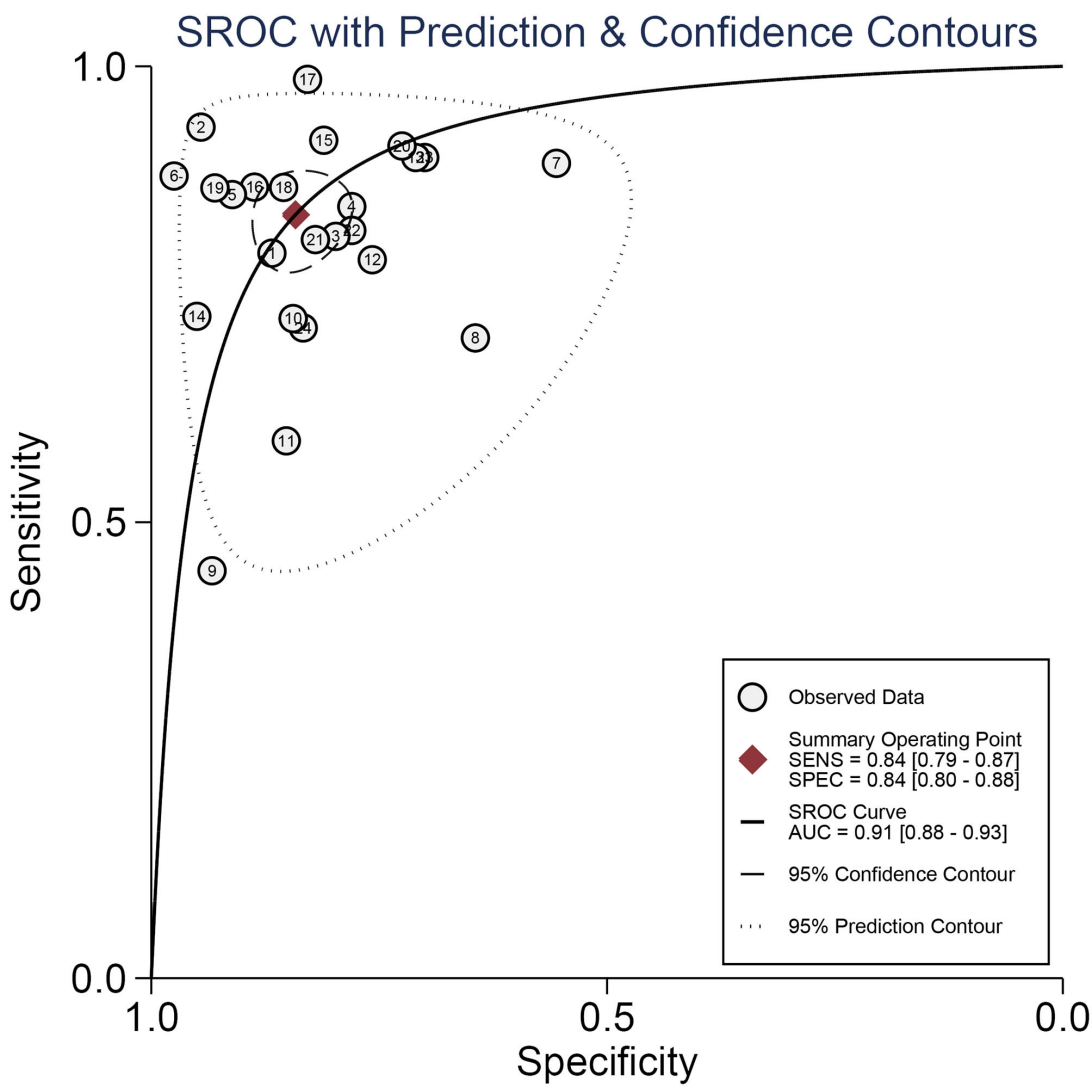
The summary receiver operating characteristic (SROC) curve of miRNAs as diagnostic biomarkers for gliomas.

**Figure 5:**
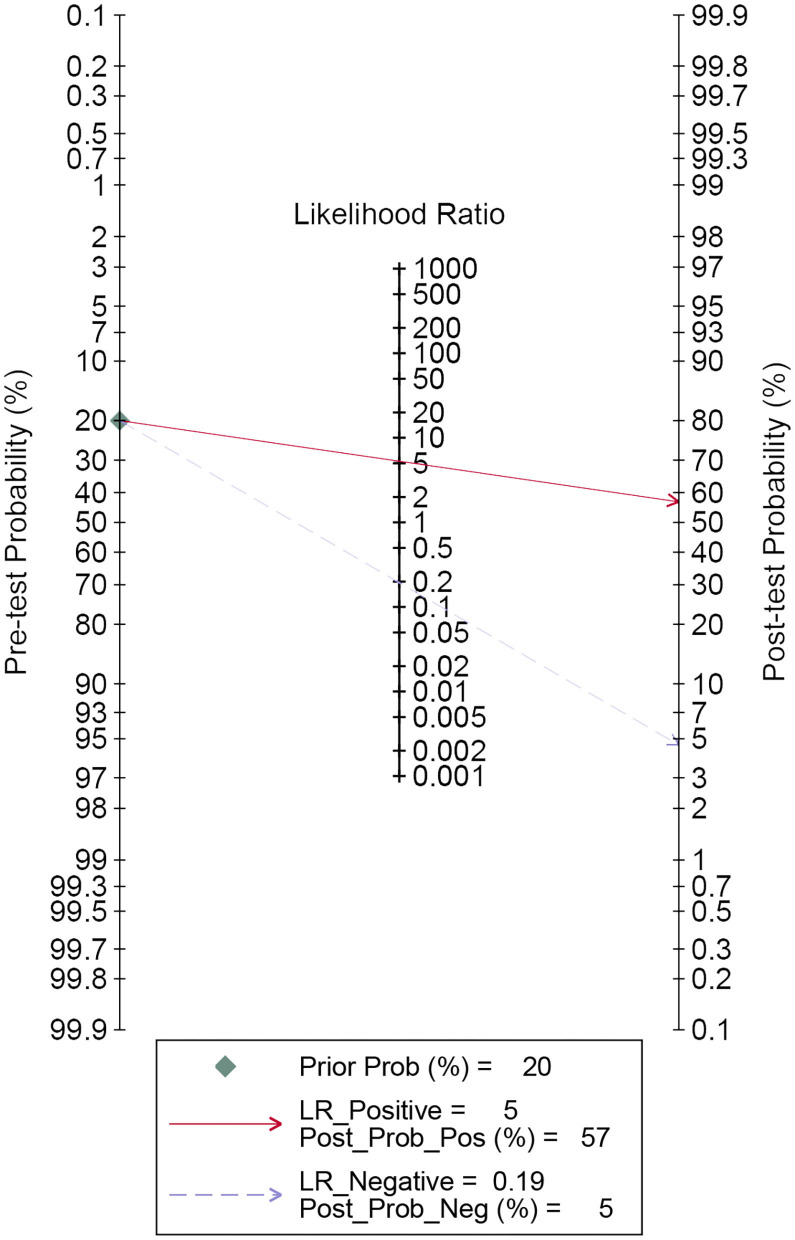
Fagan's plot for estimating post-test possibility. The red solid line denotes post-test probability when the index test is positive. While the blue dashed line indicates post-test probability with the negative index test result.

A forest plot of 24 studies on the sensitivity and specificity of miRNAs as diagnostic markers of gliomas is illustrated in Figure 3. Besides, the pooled results were listed as follows: sensitivity, 0.84 (95%CI: 0.79–0.87); specificity, 0.84 (95%CI: 0.80–0.88); positive likelihood ratio (PLR), 5.3 (95%CI: 4.1–6.8); negative likelihood ratio (NLR), 0.19 (95%CI: 0.15–0.25); and diagnostic odds ratio (DOR), 27 (95%CI: 18–41). On account of the obvious heterogeneity among studies in sensitivity (*I*^2^ = 86.06%) and specificity (*I*^2^ = 77.68%), the bivariate random-effects model was adopted to estimate the diagnostic performance of miRNAs in gliomas. Subsequently, the SROC curve was generated (Figure 4) and an AUC of 0.91 (95%CI: 0.88–0.93) was determined, which indicated the superior diagnostic value of miRNAs and showed that miRNAs could distinguish glioma patients from the healthy population. Next, a Fagan plot was applied to assess the clinical value of miRNAs in the diagnosis of gliomas. In the first place, the pre-test probability was set to 20%. Then the positive test presented a post-test probability of 57% with a PLR of 5.3. Besides, the NLR was equal to 0.19, with the negative test reducing the post-test probability to 5% (Figure 5).

### Exploring the Sources of Heterogeneity

The existence of heterogeneity attracted our attention. Firstly, we examined whether the threshold effect was at the root of heterogeneity in our study. The results indicated that there was a Spearman correlation coefficient of 0.137 (*P* = 0.525). In essence, the “shoulder arm” shape, representative shape of the threshold effect, was not found in the SROC curve. Thus, the threshold effect was excluded in the present study. Secondly, heterogeneity brought about by other factors was called the non-threshold effect. Besides, the DOR value of each study and the summary DOR value were not distributed along the same straight line, with the results of Cochran-*Q* and *I*^2^ displaying 76.87 and 70.1% (*P* = 0.0000), respectively (Supplementary Figure 1), which denoted that the non-threshold effect may have led to heterogeneity, including the sample source (plasma and serum), comparison type, the dysregulated direction of miRNAs (upregulated and downregulated), internal reference for relative quantitative analysis, miRNA profiling (single miRNA and miRNA panel), sample size (≥100 and <100), as well as cut-off values setting (given and NA).

### Subgroup Analyses, Meta-Regression, and Sensitivity Analyses

**Figure 6:**
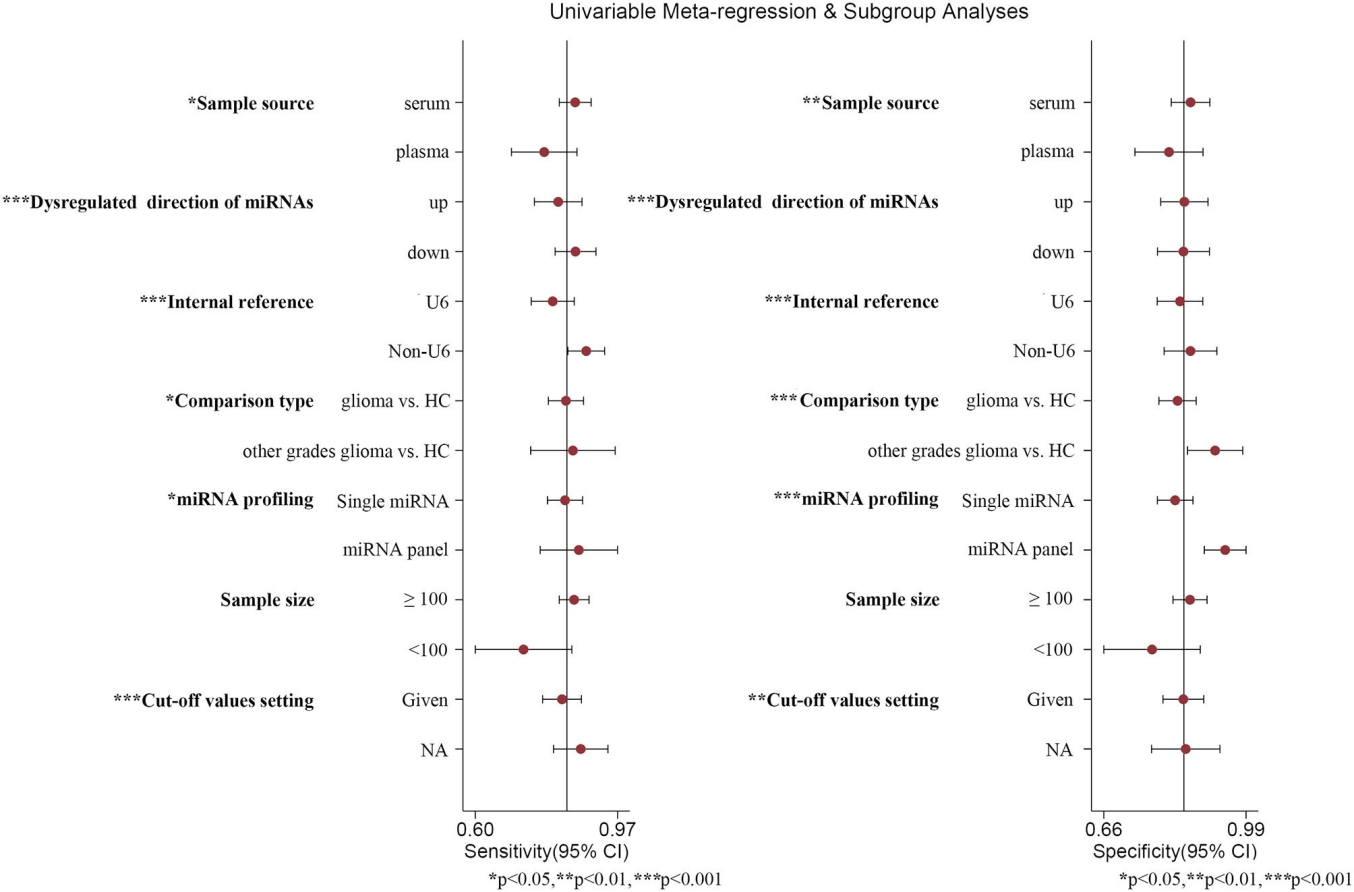
Meta-regression analysis. *, **, and *** represent *P* < 0.05, *P* < 0.01, and *P* < 0.001, respectively.

**Figure 7:**
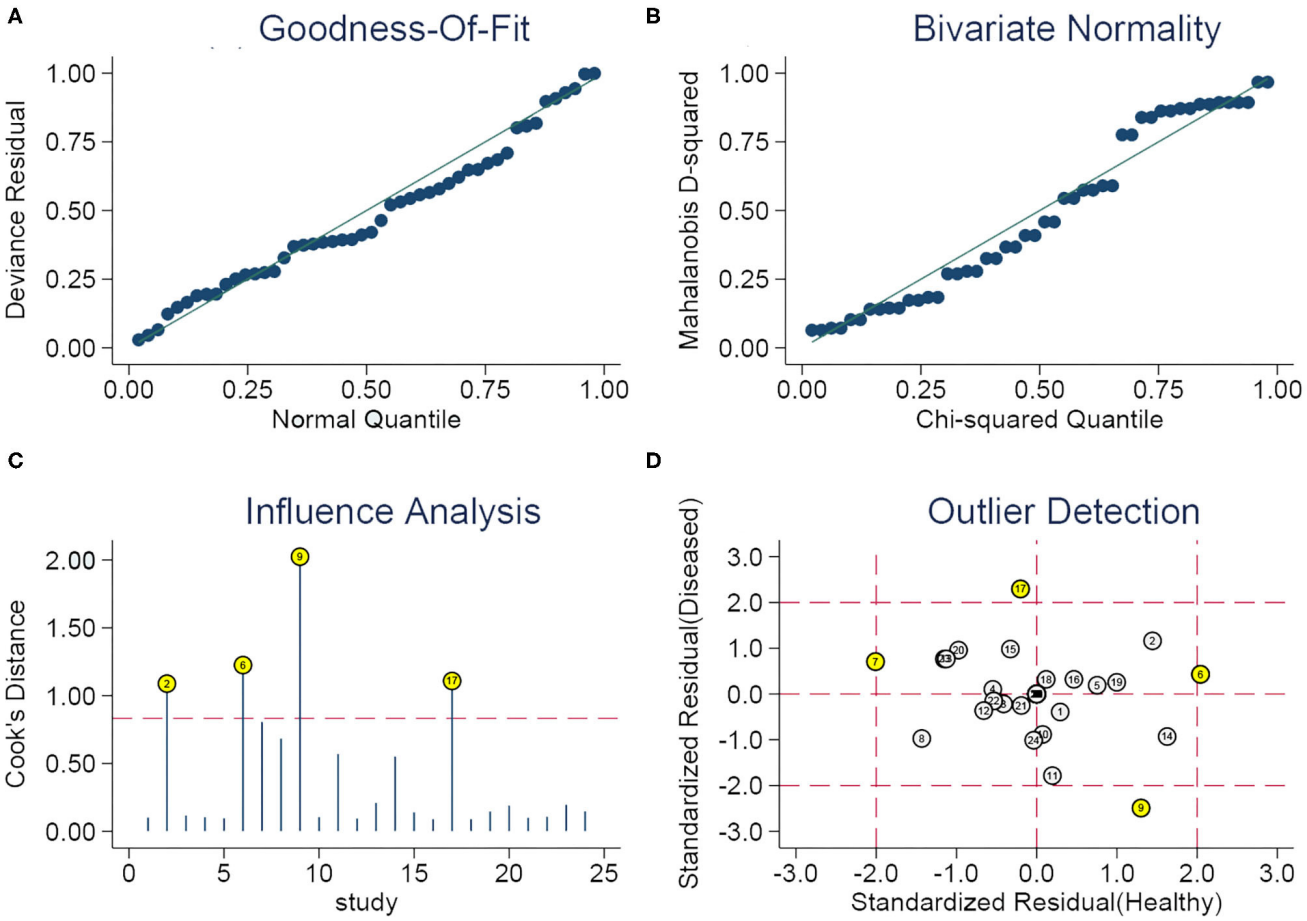
Sensitivity analysis. **(A)** Goodness of fit, **(B)** bivariate normality, **(C)** influence analysis, and **(D)** outlier detection.

Thus, we performed subgroup analyses based on the above mentioned seven subgroups (Table 3) and completed meta-regression analysis (Figure 6). The subgroups of the sample source, dysregulated direction of miRNAs, internal reference, comparison type, miRNA profiling, and cut-off values setting were related to heterogeneity (*P* < 0.05). Conversely, the difference in heterogeneity caused by a sample size subgroup was not statistically significant (*P* > 0.05). Our results indicated that serum miRNAs had superior diagnostic accuracy for glioma patients, with a sensitivity, specificity, PLR, NLR, DOR, and AUC of 0.86 (95%CI: 0.83–0.88), 0.86 (95%CI: 0.81–0.90), 6.0 (95%CI: 4.4–8.3), 0.17 (95%CI: 0.14–0.20), 36 (95%CI: 24–54), and 0.91 (95%CI: 0.88–0.93), respectively. While, the plasma-derived miRNAs in the diagnosis of gliomas had inferior performance compared to serum miRNAs and the pooled results included sensitivity (0.80, 95%CI: 0.63–0.90), specificity (0.81, 95%CI: 0.72–0.87), PLR (4.1, 95%CI: 2.9–5.9), NLR (0.25, 95%CI: 0.14–0.47), DOR (16, 95%CI: 7–36), and AUC (0.86, 95%CI: 0.83–0.89). Of note, the diagnostic efficacy of the miRNA panel was found to be rather excellent compared to a single miRNA, where the sensitivity, specificity, PLR, NLR, and DOR were 0.87 (95%CI: 0.82–0.91), 0.94 (95%CI: 0.90–0.96), 13 (95%CI: 3.7–45.5), 0.15 (95%CI: 0.06–0.36), and 96 (95%CI: 12–741), respectively. Surprisingly, miRNA expression levels were also pertinent to diagnostic value, the results denoted that upregulated miRNAs presented remarkable diagnostic efficacy (AUC: 0.90, 95%CI: 0.87–0.93). It is also of interest to note that a sample size ≥100 was closely related to better diagnostic value than a sample size of <100 cases, including sensitivity (0.86, 95%CI: 0.81–0.89), specificity (0.86, 95%CI: 0.81–0.89), PLR (5.9, 95%CI: 4.6–7.7), NLR (0.17, 95%CI: 0.13–0.22), DOR (35, 95%CI: 23–53), and AUC (0.92, 95%CI: 0.90–0.94). Additionally, the studies with optimal truncation values yielded pooled results including sensitivity, specificity, PLR, NLR, DOR, and AUC of 0.82 (95%CI: 0.77–0.87), 0.84 (95%CI: 0.79–0.88), 5.2 (95%CI: 3.8–7.0), 0.21 (95%CI: 0.16–0.28), 25 (95%CI: 15–40), and 0.90 (95%CI: 0.87–0.92), whereas the studies without cut-off values presented preferable diagnostic value, with sensitivity of 0.87 (95%CI: 0.79–0.93), specificity of 0.85 (95%CI: 0.75–0.91), PLR of 5.6 (95%CI: 3.5–9.2), NLR of 0.15 (95%CI: 0.09–0.25), DOR of 37 (95%CI: 19–73), and AUC of 0.92 (95%CI: 0.90–0.94), respectively (Table 2). Furthermore, we also conducted a goodness-of-fit analysis and bivariate normality analysis (Figures 7A,B), showing that our model was robust. The outliers were detected via sensitivity analysis, with a total of four studies deemed to be outliers, including Yang et al., Xu et al. (miR-17 and miR-10b), and Shao et al. (Figures 7C,D). After removing these four outliers, the pooled results were found to have no significant alterations (Table 4).

**Table 3:** The results of subgroup analysis.

**Covariates**	**No. studies**	**Sen (95%CI)**	**Spe (95%CI)**	**PLR (95%CI)**	**NLR (95%CI)**	**DOR (95%CI)**	**AUC (95%CI)**
**Sample source**							
Plasma	8	0.80 (0.63–0.90)	0.81 (0.72–0.87)	4.1 (2.9–5.9)	0.25 (0.14–0.47)	16 (7–36)	0.86 (0.83–0.89)
Serum	16	0.86 (0.83–0.88)	0.86 (0.81–0.90)	6.0 (4.4–8.3)	0.17 (0.14–0.20)	36 (24–54)	0.91 (0.88–0.93)
**Dysregulated miRNAs**							
Up	13	0.82 (0.73–0.89)	0.84 (0.77–0.90)	5.3 (3.6–7.8)	0.21 (0.14–0.33)	25 (13–47)	0.90 (0.87–0.93)
Down	11	0.85 (0.83–0.87)	0.84 (0.78–0.88)	5.3 (3.9–7.2)	0.17 (0.15–0.21)	30 (20–46)	0.88 (0.85–0.90)
**Internal reference**							
U6	15	0.80 (0.74–0.86)	0.83 (0.77–0.88)	4.8 (3.5–6.7)	0.24 (0.18–0.31)	20 (13–32)	0.89 (0.86–0.91)
Non–U6	7	0.86 (0.83–0.88)	0.83 (0.77–0.88)	5.1 (3.7–7.1)	0.17 (0.14–0.21)	30 (19– 47)	0.88 (0.85–0.91)
NA	2	0.96 (0.91–0.98)	0.90 (0.85–0.94)	9.6 (3.2–29)	0.05 (0.01–0.17)	262 (95–724)	NA^a^
**Comparison type**							
Gliomas vs. HC	21	0.84 (0.78–0.88)	0.83 (0.78–0.87)	4.8 (3.8–6.1)	0.20 (0.15–0.26)	24 (16–36)	0.90 (0.87–0.92)
HGG vs. HC	2	0.80 (0.74–0.86)	0.89 (0.83–0.94)	7.1 (1.9–26.5)	0.24 (0.17–0.35)	30 (12–74)	NA^a^
Astrocytomas vs. HC	1	0.933	0.945	–	–	–	0.97 (0.95–0.99)
**miRNA profiling**							
Single miRNA	21	0.83 (0.78–0.87)	0.82 (0.78–0.86)	4.7 (3.8–5.8)	0.20 (0.16–0.26)	23 (16–32)	0.89 (0.86–0.92)
miRNA panel	3	0.87 (0.82–0.91)	0.94 (0.90 −0.96)	13 (3.7–45.5)	0.15 (0.06–0.36)	96 (12–741)	NA^a^
**Sample size**							
≥100	19	0.86 (0.81–0.89)	0.86 (0.81–0.89)	5.9 (4.6–7.7)	0.17 (0.13–0.22)	35 (23–53)	0.92 (0.90–0.94)
<100	5	0.73 (0.58–0.84)	0.77 (0.61–0.88)	3.2 (2.0–5.0)	0.36 (0.25–0.51)	9 (6–14)	0.81 (0.78–0.84)
**Cut-off values**							
Given	18	0.82 (0.77–0.87)	0.84 (0.79–0.88)	5.2 (3.8–7.0)	0.21 (0.16–0.28)	25 (15–40)	0.90 (0.87–0.92)
NA	6	0.87 (0.79–0.93)	0.85 (0.75–0.91)	5.6 (3.5–9.2)	0.15 (0.09–0.25)	37 (19–73)	0.92 (0.90–0.94)

a *The number of studies was too small to fit the curve*.

*Sen, sensitivity; Spe, specificity; PLR, positive likelihood ratio; NLR, negative likelihood ratio; DOR, diagnostic odds ratio; AUC, area under the curve; NA, not available; CI, confidence interval*.

**Table 4:** Diagnostic performance of miRNAs in gliomas.

**Analysis**	**Overall**	**Outliers excluded**
No. of studies	24	20
Sen (95% CI)	0.84 (0.79–0.87)	0.83 (0.79–0.87)
Spe (95% CI)	0.84 (0.80–0.88)	0.83 (0.79–0.87)
PLR (95% CI)	5.3 (4.1–6.8)	5.0 (4.0–6.3)
NLR (95% CI)	0.19 (0.15–0.25)	0.20 (0.16–0.25)
DOR (95% CI)	27 (18–41)	25 (17–36)
AUC (95% CI)	0.91 (0.88–0.93)	0.90 (0.87–0.92)

*Sen, sensitivity; Spe, specificity; PLR, positive likelihood ratio; NLR, negative likelihood ratio; DOR, diagnostic odds ratio; AUC, area under the curve; CI, confidence interval*.

### Publication Bias

**Figure 8:**
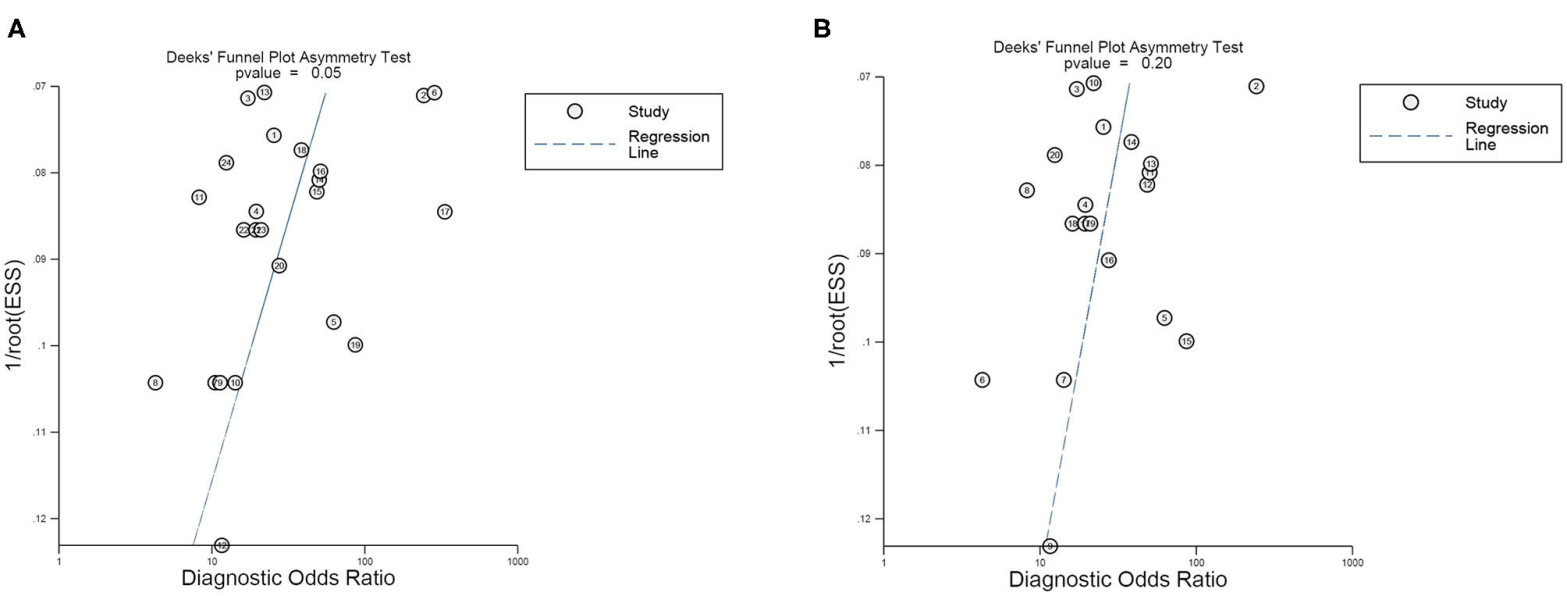
Deek's funnel plot asymmetry test for the evaluation of publication bias of **(A)** all 24 studies included and **(B)** 20 studies after eliminating the four outliers.

Subsequently, we implemented a Deek's funnel plot asymmetry test, as well as a line regression test. Asymmetry (Figure 8A) was observed and the results of the linear regression test were −2.07 (*P* = 0.05), indicating the existence of publication bias in our meta-analysis. Nevertheless, after removing the four outliers, the Deek's funnel plot indicated that publication bias was not found (Figure 8B, *t* = −1.35, *P* = 0.20).

## Discussion

In the present study, a total of 18 articles covering 24 studies included 2,170 glioma patients and 1,456 healthy individuals, with the results denoting that the pooled sensitivity, specificity, PLR, NLR, DOR, and AUC of miRNAs in the diagnosis of gliomas were 0.84 (95%CI: 0.79–0.87), 0.84 (95%CI: 0.80–0.88), 5.3 (95%CI: 4.1–6.8), 0.19 (95%CI: 0.15–0.25), 27 (95%CI: 18–41), and 0.91 (95%CI: 0.88–0.93), respectively. A pooled PLR of 5.3 referred to the probability of individuals diagnosed with gliomas increasing by 5.3-fold when an miRNA test was positive. Additionally, the value of NLR (0.19) was reflective of only a 19% probability of subjects diagnosed with gliomas if miRNA detection was negative. DOR, an index of discriminatory test performance (61), was employed to assess the discrimination effect of miRNAs in the diagnosis of gliomas, which gave a value of 27 (>1.0), indicating that the circulating miRNAs can effectively differentiate the glioma patients from the healthy population. Qu et al. (62) explored the efficacy of miRNAs as diagnostic markers of gliomas via a meta-analysis and they concluded similar results as follows: sensitivity, 0.87 (95% CI: 0.83–0.91); specificity, 0.87 (95% CI: 0.81–0.91); PLR, 6.6 (95% CI: 4.5–9.6), NLR, 0.15 (95% CI: 0.10–0.21); DOR, 45 (95% CI: 23–90); and AUC, 0.93 (95% CI: 0.91–0.95), indicating the potential diagnostic value of miRNAs for the glioma population. However, subgroup analyses for exploring the sources of heterogeneity investigated miRNA profiling, but other factors were neglected. In the present study, through subgroup and meta-regression analyses, the factors influencing heterogeneity contained sample source, dysregulated direction of miRNAs, internal reference, comparison type, miRNAs profiling, as well as cut-off values setting (*P* < 0.05). We found that serum miRNAs presented remarkable diagnostic efficacy for gliomas with a sensitivity, specificity, and AUC of 0.86 (95%CI: 0.83–0.88), 0.86 (95%CI: 0.81–0.90), and 0.91 (95%CI: 0.88–0.93), respectively, indicating their promising diagnostic ability for glioma patients. In a previous study, Wang et al. (54) revealed that serum miR-214 gradually escalated with an increase in glioma grade, and had excellent diagnostic efficacy for gliomas (AUC: 0.885, 95%CI: 0.833–0.926). Additionally, Lai et al. (49) showed the outstanding diagnostic value of miR-210 in gliomas with a sensitivity of 0.9127, specificity of 0.725, and AUC of 0.927 (95%CI: 0.889–0.964). Also, Lan et al. (28) proposed that serum exosomal miR-301a could act as a diagnostic biomarker of a glioma and the results were as follows: sensitivity, 0.862; specificity, 0.932; AUC, 0.937 (95%CI: 0.855–0.987). Likewise, Shao et al. (51) constructed a ROC curve for revealing the capability of miR-454-3p in discriminating glioma patients from healthy participants. At the optimal cut-off value of ROC curve, the sensitivity, specificity, and AUC of miR-454-3p were 0.9905, 0.8286, and 0.9063 (95%CI: 0.8487–0.9639), respectively. Sun et al. (52) demonstrated that miR-128 could reliably distinguish glioma patients from healthy individuals, with an AUC of 0.9095 (95%CI: 0.8695–0.9496), and at an optimum cut-off value of 7.139, the sensitivity and specificity were 0.8675 and 0.8868, respectively. Considering that all the studies applied the qRT-PCR method to detect the miRNAs levels, it is necessary to choose a suitable internal reference for normalization, of which U6 is the most common. When U6 was selected as internal reference for relative quantitative analysis, we found that miRNAs showed a similar diagnostic accuracy with other materials known as non-U6 references. Conversely, Xiang et al. (63) found that U6 was not appropriate as an internal reference for miRNA quantitative detection standardization due to its instability. Thus, for the better application of miRNAs in clinical practice as diagnostic biomarkers, it is extremely important to select a unified, suitable, and stable internal reference in the future. In addition, an miRNA panel may be more suitable as a diagnostic biomarker for gliomas, which is the future trend of development on account of miRNAs functioning as a significant sub-class of non-coding RNA. Similarly to our findings, Xu et al. demonstrated that a three plasma-derived miRNA signature had the best diagnostic performance over any other single miRNA, with an AUC of 0.872 (95%CI: 0.78–0.93) (35). In a previous study, Yang et al. (56) conducted serum miRNA profiling analysis, validated seven miRNAs via the quantitative reverse-transcription PCR (RT-qPCR) method, and found that the seven serum miRNA signature had outstanding sensitivity (0.88), specificity (0.9787), and AUC (0.972, 95%CI: 0.954–0.990). Moreover, Zhi et al. (34) established a 9-miRNA panel for evaluating its diagnostic value for astrocytomas, with extraordinary sensitivity (0.933), specificity (0.945), and AUC (0.9722, 95%CI: 0.9501–0.9942). Intriguingly, a higher diagnostic value in the elevated miRNA group was observed, which may be attributed to the fact that upregulated miRNAs were more easily detected than the downregulated group. Additionally, a sample size ≥100 was associated with good diagnostic efficacy, which was demonstrated by Zhi et al. (34), Yang et al. (56), Sun et al. (52), and so on. Thus, a large sample size and large-scale studies will be needed to verify these findings. In addition, for clinical use, a Fagan plot was used for exploring miRNAs as diagnostic markers for estimating the possibility of individuals who would be diagnosed with a glioma, which indicated promising results. When it comes to the detection methods of miRNAs, all studies adopted the qRT-PCR method, which can reverse transcribe an RNA strand into a complementary DNA (cDNA) chain and this cDNA chain will act as a template for the accomplishment of DNA amplification. Therefore, methods for the detection of miRNAs were not taken into account in the subgroup and meta-regression analyses.

However, limitations also need to be highlighted in our meta-analysis. Firstly, due to these studies belonging to retrospective research, a lack of prospective studies increased the bias risk of quality assessment in the patient selection and index test domains. Secondly, there was no consensus about the unified internal reference, leading to inconsistent results in miRNA relative quantitative analysis. Thirdly, the conventional and standard method of miRNA detection was the qRT-PCR method, characterized by complicated, tedious, and time-consuming manipulation (64), high experimental requirements, and the use of RNA which easily degrades, which limited extensive clinical practice (65). Hence, some novel methods need to be developed for implementing rapid miRNA detection with high sensitivity and selectivity, such as a toehold-mediated strand displacement reaction (SDR) (64), the enzyme-free surface plasmon resonance imaging (SPRi) biosensing method (66), and the ultrasensitive electrochemical method (67), etc. Moreover, the ethnicity of all participants was Asian and they all came from China, with miRNAs showing outstanding diagnostic value for gliomas, but its diagnostic performance may not be suitable for glioma patients worldwide. Additionally, publication bias existed in the present study, but after eliminating the four outliers via sensitivity analysis, the publication bias was found to not be significant, which may be pertinent to the lack of studies with negative results, heterogeneity between-studies, as well as the quality of the included studies. In addition, based on the clinical and pathological characteristics of participants in the included studies, the heterogeneity of tumor types was the inherent limitation of the field. Furthermore, due to a lack of investigation into the diagnostic efficacy of miRNAs in the LGG, the present study lacked the evidence of miRNAs as diagnostic markers for identifying gliomas at an early stage. Ultimately, on account of the absence of similar miRNAs to pool the results, specific miRNAs could not be identified as diagnostic biomarkers for gliomas, which is contrary to our original intention. Therefore, based on the limitations mentioned above, these findings need to be carefully interpreted.

Taken together, the remarkable diagnostic efficacy of circulating miRNAs, especially upregulated serum miRNAs, has gradually emerged for the glioma population. Specifically, an miRNA panel had superior diagnostic value when compared to single miRNA in the diagnosis of gliomas. Nevertheless, a large pool of prospective studies and multi-center research will be required to confirm our findings in the near future. In the next step, we will collect blood samples from LGG patients for an investigation into the potential diagnostic performance of miRNAs and verifying its pivotal functions in early diagnosis.

## Data Availability Statement

The original contributions presented in the study are included in the article/Supplementary Material, further inquiries can be directed to the corresponding author/s.

## Author Contributions

JH and YJ designed the project, constructed the search strategy, screened the articles, and drafted the manuscript. JH and CZ extracted the data of the studies included. YJ and ZZ performed the quality assessment and revised the figures. JH, LL, and CZ implemented the data analysis. LL and CZ revised the manuscript and interpreted the results. All authors contributed to the article and approved the submitted version.

## Conflict of Interest

The authors declare that the research was conducted in the absence of any commercial or financial relationships that could be construed as a potential conflict of interest.

## References

[1] Ostrom Q Cioffi G Gittleman H Kruchko C Waite K Barnholtz-Sloan J. CBTRUS statistical report: primary brain and other central nervous system tumors diagnosed in the United States in 2012-2016. Neuro Oncol. (2019) 21:v1–100. 10.1093/neuonc/noz150 31675094 PMC6823730

[2] Ostrom QT Gittleman H Farah P Ondracek A Chen Y Wolinsky Y. CBTRUS statistical report: primary brain and central nervous system tumors diagnosed in the United States in 2006-2010. Neuro Oncol. (2013) 15:i1–56. 10.1093/neuonc/not151 24137015 PMC3798196

[3] Ostrom QT Bauchet L Davis FG Deltour I Fisher JL Langer CE. The epidemiology of glioma in adults: a “state of the science” review. Neuro Oncol. (2014) 16:896–913. 10.1093/neuonc/nou087 24842956 PMC4057143

[4] Van Meir EG Hadjipanayis CG Norden AD Shu HK Wen PY Olson JJ. Exciting new advances in neuro-oncology: the avenue to a cure for malignant glioma. CA Cancer J Clin. (2010) 60:166–93. 10.3322/caac.20069 20445000 PMC2888474

[5] Louis DN Ohgaki H Wiestler OD Cavenee WK Burger PC Jouvet A. The 2007 WHO classification of tumours of the central nervous system. Acta Neuropathol. (2007) 114:97–109. 10.1007/s00401-007-0243-4 17618441 PMC1929165

[6] Louis DN Perry A Reifenberger G von Deimling A Figarella-Branger D Cavenee WK et al. The 2016 World Health Organization classification of tumors of the central nervous system: a summary. Acta Neuropathol. (2016) 131:803–20. 10.1007/s00401-016-1545-1 27157931

[7] Smoll NR Gautschi OP Schatlo B Schaller K Weber DC. Relative survival of patients with supratentorial low-grade gliomas. Neuro Oncol. (2012) 14:1062–9. 10.1093/neuonc/nos144 22773277 PMC3408266

[8] Stupp R Mason WP van den Bent MJ Weller M Fisher B Taphoorn MJ. Radiotherapy plus concomitant and adjuvant temozolomide for glioblastoma. N Engl J Med. (2005) 352:987–96. 10.1056/NEJMoa043330 15758009

[9] Mabray MC Barajas RF Cha S. Modern brain tumor imaging. Brain Tumor Res Treat. (2015) 3:8–23. 10.14791/btrt.2015.3.1.8 25977902 PMC4426283

[10] Regazzo G Terrenato I Spagnuolo M Carosi M Cognetti G Cicchillitti L. A restricted signature of serum miRNAs distinguishes glioblastoma from lower grade gliomas. J Exp Clin Cancer Res. (2016) 35:124. 10.1186/s13046-016-0393-0 27476114 PMC4967504

[11] Westphal M Lamszus K. Circulating biomarkers for gliomas. Nat Rev Neurol. (2015) 11:556–66. 10.1038/nrneurol.2015.171 26369507

[12] Kros JM Mustafa DM Dekker LJ Sillevis Smitt PA Luider TM Zheng PP. Circulating glioma biomarkers. Neuro Oncol. (2015) 17:343–60. 10.1093/neuonc/nou207 25253418 PMC4483097

[13] Berindan-Neagoe I Monroig Pdel C Pasculli B Calin GA. MicroRNAome genome: a treasure for cancer diagnosis and therapy. CA Cancer J Clin. (2014) 64:311–36. 10.3322/caac.21244 25104502 PMC4461198

[14] He L Hannon GJ. MicroRNAs: small RNAs with a big role in gene regulation. Nat Rev Genet. (2004) 5:522–31. 10.1038/nrg1379 15211354

[15] Croce CM. Causes and consequences of microRNA dysregulation in cancer. Nat Rev Genet. (2009) 10:704–14. 10.1038/nrg2634 19763153 PMC3467096

[16] Garzon R Marcucci G Croce CM. Targeting microRNAs in cancer: rationale, strategies and challenges. Nat Rev Drug Discov. (2010) 9:775–89. 10.1038/nrd3179 20885409 PMC3904431

[17] He L Thomson JM Hemann MT Hernando-Monge E Mu D Goodson S. A microRNA polycistron as a potential human oncogene. Nature. (2005) 435:828–33. 10.1038/nature03552 15944707 PMC4599349

[18] Huang Q Gumireddy K Schrier M le Sage C Nagel R Nair S. The microRNAs miR-373 and miR-520c promote tumour invasion and metastasis. Nat Cell Biol. (2008) 10:202–10. 10.1038/ncb1681 18193036

[19] Hatfield SD Shcherbata HR Fischer KA Nakahara K Carthew RW Ruohola-Baker H. Stem cell division is regulated by the microRNA pathway. Nature. (2005) 435:974–8. 10.1038/nature03816 15944714

[20] Choi HH Cho YS Choi JH Kim HK Kim SS Chae HS. Stool-based miR-92a and miR-144^*^ as noninvasive biomarkers for colorectal cancer screening. Oncology. (2019) 97:173–9. 10.1159/000500639 31216561

[21] Sun Y Yang B Lin M Yu H Chen H Zhang Z. Identification of serum miR-30a-5p as a diagnostic and prognostic biomarker in colorectal cancer. Cancer Biomark. (2019) 24:299–305. 10.3233/CBM-182129 30829615 PMC13082511

[22] Huang C Wang Q Ma S Sun Y Vadamootoo AS Jin C. A four serum-miRNA panel serves as a potential diagnostic biomarker of osteosarcoma. Int J Clin Oncol. (2019) 24:976–82. 10.1007/s10147-019-01433-x 31111286

[23] Fan C Liu N. Identification of dysregulated microRNAs associated with diagnosis and prognosis in triple-negative breast cancer: an *in silico* study. Oncol Rep. (2019) 41:3313–24. 10.3892/or.2019.7094 30942465

[24] Orangi E Motovali-Bashi M. Evaluation of miRNA-9 and miRNA-34a as potential biomarkers for diagnosis of breast cancer in Iranian women. Gene. (2019) 687:272–9. 10.1016/j.gene.2018.11.036 30468908

[25] Moya L Meijer J Schubert S Matin F Batra J. Assessment of miR-98-5p, miR-152-3p, miR-326 and miR-4289 expression as biomarker for prostate cancer diagnosis. Int J Mol Sci. (2019) 20:1154–173. 10.3390/ijms20051154 30845775 PMC6429489

[26] Shi C Yang Y Zhang L Zhang T Yu J Qin S. Optimal subset of signature miRNAs consisting of 7 miRNAs that can serve as a novel diagnostic and prognostic predictor for the progression of cervical cancer. Oncol Rep. (2019) 41:3167–78. 10.3892/or.2019.7097 30942460 PMC6489013

[27] Zhang H Wang J Wang Z Ruan C Wang L Guo H. Serum miR-100 is a potential biomarker for detection and outcome prediction of glioblastoma patients. Cancer Biomark. (2019) 24:43–9. 10.3233/CBM-181416 30530966 PMC13082502

[28] Lan F Qing Q Pan Q Hu M Yu H Yue X. Serum exosomal miR-301a as a potential diagnostic and prognostic biomarker for human glioma. Cell Oncol. (2018) 41:25–33. 10.1007/s13402-017-0355-3 29076027 PMC12995224

[29] Yang L Ma Y Xin Y Han R Li R Hao X. Role of the microRNA 181 family in glioma development. Mol Med Rep. (2018) 17:322–9. 10.3892/mmr.2017.7895 29115595

[30] Kosaka N Ochiya T. Unraveling the mystery of cancer by secretory microRNA: horizontal microRNA transfer between living cells. Front Genet. (2011) 2:97. 10.3389/fgene.2011.00097 22303391 PMC3262223

[31] Solayman MH Langaee T Patel A Elwakeel L Elhamamsy M Badary O. Identification of suitable endogenous normalizers for qRT-PCR analysis of plasma microRNA expression in essential hypertension. Mol Biotechnol. (2016) 58:179–87. 10.1007/s12033-015-9912-z 26798072 PMC4758859

[32] Sanz-Rubio D Martin-Burriel I Gil A Cubero P Forner M Khalyfa A. Stability of circulating exosomal miRNAs in healthy subjects. Sci Rep. (2018) 8:10306. 10.1038/s41598-018-28748-5 29985466 PMC6037782

[33] Ye X Wei W Zhang Z He C Yang R Zhang J. Identification of microRNAs associated with glioma diagnosis and prognosis. Oncotarget. (2017) 8:26394–403. 10.18632/oncotarget.14445 28060761 PMC5432266

[34] Zhi F Shao N Wang R Deng D Xue L Wang Q. Identification of 9 serum microRNAs as potential noninvasive biomarkers of human astrocytoma. Neuro-Oncology. (2015) 17:383–91. 10.1093/neuonc/nou169 25140035 PMC4483096

[35] Xu W Liang W Dai Y. A three-miRNA signature as a potential biomarker for the diagnosis of glioma. Int J Clin Exp Pathol. (2017) 10:2814–23. 31909898

[36] Xiao Y Zhang L Song Z Guo C Zhu J Li Z. Potential diagnostic and prognostic value of plasma circulating MicroRNA-182 in human glioma. Med Sci Monit. (2016) 22:855–62. 10.12659/MSM.897164 26978735 PMC4795091

[37] Whiting PF Rutjes AW Westwood ME Mallett S Deeks JJ Reitsma JB QUADAS-2: A revised tool for the quality assessment of diagnostic accuracy studies. Ann Intern Med. (2011) 115:529–36. 10.7326/0003-4819-155-8-201110180-00009 22007046

[38] Wade R Corbett M Eastwood A. Quality assessment of comparative diagnostic accuracy studies: our experience using a modified version of the QUADAS-2 tool. Res Synth Methods. (2013) 4:280–6. 10.1002/jrsm.1080 26053845

[39] Moody L Dvoretskiy S An R Mantha S Pan YX. The efficacy of miR-20a as a diagnostic and prognostic biomarker for colorectal cancer: a systematic review and meta-analysis. Cancers. (2019) 11:1111–124. 10.3390/cancers11081111 31382594 PMC6721456

[40] Higgins JP Thompson SG Deeks JJ Altman DG. Measuring inconsistency in meta-analyses. BMJ. (2003) 327:557–60. 10.1136/bmj.327.7414.557 12958120 PMC192859

[41] Arends LR Hamza TH van Houwelingen JC Heijenbrok-Kal MH Hunink MG Stijnen T. Bivariate random effects meta-analysis of ROC curves. Med Decis Making. (2008) 28:621–38. 10.1177/0272989X08319957 18591542

[42] Reitsma JB Glas AS Rutjes AW Scholten RJ Bossuyt PM Zwinderman AH. Bivariate analysis of sensitivity and specificity produces informative summary measures in diagnostic reviews. J Clin Epidemiol. (2005) 58:982–90. 10.1016/j.jclinepi.2005.02.022 16168343

[43] Kotz S Balakrishnan N Johnson NL Bivariate and trivariate normal distributions. In: Continuous Multivariate Distributions. New York, NY: Wiley (2000). p. 251–348. 10.1002/0471722065

[44] Zamora J Abraira V Muriel A Khan K Coomarasamy A. Meta-DiSc: a software for meta-analysis of test accuracy data. BMC Med Res Methodol. (2006) 6:31. 10.1186/1471-2288-6-31 16836745 PMC1552081

[45] Wu F Huang Y Huang X. 99mTc-MIBI scintigraphy for the preoperative assessment of histological response to neoadjuvant chemotherapy in patients with osteosarcoma: a systematic review and a bivariate meta-analysis. Front Oncol. (2020) 10:762. 10.3389/fonc.2020.00762 32528883 PMC7258398

[46] Deeks JJ Macaskill P Irwig L. The performance of tests of publication bias and other sample size effects in systematic reviews of diagnostic test accuracy was assessed. J Clin Epidemiol. (2005) 58:882–93. 10.1016/j.jclinepi.2005.01.016 16085191

[47] Chen P Zhang G Zhou Q Li Z. Plasma microRNA-720 may predict prognosis and diagnosis in glioma patients. Biosci Rep. (2020) 40:BSR20201449. 10.1042/BSR20201449 32639004 PMC7364510

[48] Huang Q Wang C Hou Z Wang G Lv J Wang H. Serum microRNA-376 family as diagnostic and prognostic markers in human glioma. Cancer Biomark. (2017) 19:137–44. 10.3233/CBM-160146 28211798 PMC13020711

[49] Lai NS Wu DG Fang XG Lin YC Chen SS Li ZB. Serum microRNA-210 as a potential noninvasive biomarker for the diagnosis and prognosis of glioma. Br J Cancer. (2015) 112:1241–6. 10.1038/bjc.2015.91 25756397 PMC4385967

[50] Qi Y Gao Y. Clinical significance of miR-33b in glioma and its regulatory role in tumor cell proliferation, invasion and migration. Biomark Med. (2020) 14:539–48. 10.2217/bmm-2019-0455 32462908

[51] Shao N Wang L Xue L Wang R Lan Q. Plasma miR-454-3p as a potential prognostic indicator in human glioma. Neurol Sci. (2015) 36:309–13. 10.1007/s10072-014-1938-7 25190548

[52] Sun J Liao K Wu X Huang J Zhang S Lu X. Serum microRNA-128 as a biomarker for diagnosis of glioma. Int J Clin Exp Med. (2015) 8:456–63. 25785017 PMC4358472

[53] Tang Y Zhao S Wang J Li D Ren Q Tang Y. Plasma miR-122 as a potential diagnostic and prognostic indicator in human glioma. Neurol Sci. (2017) 38:1087–92. 10.1007/s10072-017-2912-y 28367610

[54] Wang J Che F Zhang J Zhang M Xiao S Liu Y. Diagnostic and Prognostic potential of serum cell-free microRNA-214 in glioma. World Neurosurg. (2019) 125:e1217–25. 10.1016/j.wneu.2019.02.009 30794970

[55] Wei X Chen D Lv T Li G Qu S. Serum MicroRNA-125b as a potential biomarker for glioma diagnosis. Mol Neurobiol. (2016) 53:163–70. 10.1007/s12035-014-8993-1 25416859

[56] Yang C Wang C Chen X Chen S Zhang Y Zhi F. Identification of seven serum microRNAs from a genome-wide serum microRNA expression profile as potential noninvasive biomarkers for malignant astrocytomas. Int J Cancer. (2012) 132:116–27. 10.1002/ijc.27657 22674182

[57] Yue X Lan F Hu M Pan Q Wang Q Wang J. Downregulation of serum microRNA-205 as a potential diagnostic and prognostic biomarker for human glioma. J Neurosurg. (2016) 124:122–8. 10.3171/2015.1.JNS141577 26230475

[58] Zhang Y Ta WW Sun PF Meng YF Zhao CZ. Diagnostic and prognostic significance of serum miR-145-5p expression in glioblastoma. Int J Clin Exp Pathol. (2019) 12:2536–43. 31934080 PMC6949540

[59] Zhao SP Yue SZ Yang QH Cai WM Jin CL Gao GJ Serum microRNA-451a expression and its diagnostic value in glioma. Int J Clin Exp Pathol. (2016) 9:3678-82

[60] Zhu M Zhao W Zhao H Zhang J. Diagnostic and prognostic value of microRNA-193b in patients with glioma and its effect on tumor progression. Oncol Lett. (2019) 18:4882–90. 10.3892/ol.2019.10819 31611998 PMC6781758

[61] Glas AS Lijmer JG Prins MH Bonsel GJ Bossuyt PM. The diagnostic odds ratio: a single indicator of test performance. J Clin Epidemiol. (2003) 56:1129–35. 10.1016/S0895-4356(03)00177-X 14615004

[62] Qu S Guan J Liu Y. Identification of microRNAs as novel biomarkers for glioma detection: a meta-analysis based on 11 articles. J Neurol Sci. (2015) 348:181–7. 10.1016/j.jns.2014.11.036 25510379

[63] Xiang M Zeng Y Yang R Xu H Chen Z Zhong J. U6 is not a suitable endogenous control for the quantification of circulating microRNAs. Biochem Biophys Res Commun. (2014) 454:210–4. 10.1016/j.bbrc.2014.10.064 25450382

[64] Li W Jiang W Ding Y Wang L. Highly selective and sensitive detection of miRNA based on toehold-mediated strand displacement reaction and DNA tetrahedron substrate. Biosens Bioelectron. (2015) 71:401–6. 10.1016/j.bios.2015.04.067 25950935

[65] Furlan I Domljanovic I Uhd J Astakhova K. Improving the design of synthetic oligonucleotide probes by fluorescence melting assay. Chembiochem. (2019) 20:587–94. 10.1002/cbic.201800511 30211970

[66] Wei X Liu D Zhao M Yang T Fan Y Chen W. An enzyme-free surface plasmon resonance imaging biosensing method for highly sensitive detection of microRNA based on catalytic hairpin assembly and spherical nucleic acid. Anal Chim Acta. (2020) 1108:21–7. 10.1016/j.aca.2020.02.055 32222240

[67] Liu L Song C Zhang Z Yang J Zhou L Zhang X. Ultrasensitive electrochemical detection of microRNA-21 combining layered nanostructure of oxidized single-walled carbon nanotubes and nanodiamonds by hybridization chain reaction. Biosens Bioelectron. (2015) 70:351–7. 10.1016/j.bios.2015.03.051 25841119

