# Supplementary figures and images for: Circulating MicroRNAs as Promising Diagnostic Biomarkers for Patients With Glioma: A Meta-Analysis
Source: Front Neurol. 2021 Feb 1;11:610163. doi: 10.3389/fneur.2020.610163 (PMC7882507; doi:10.3389/fneur.2020.610163)

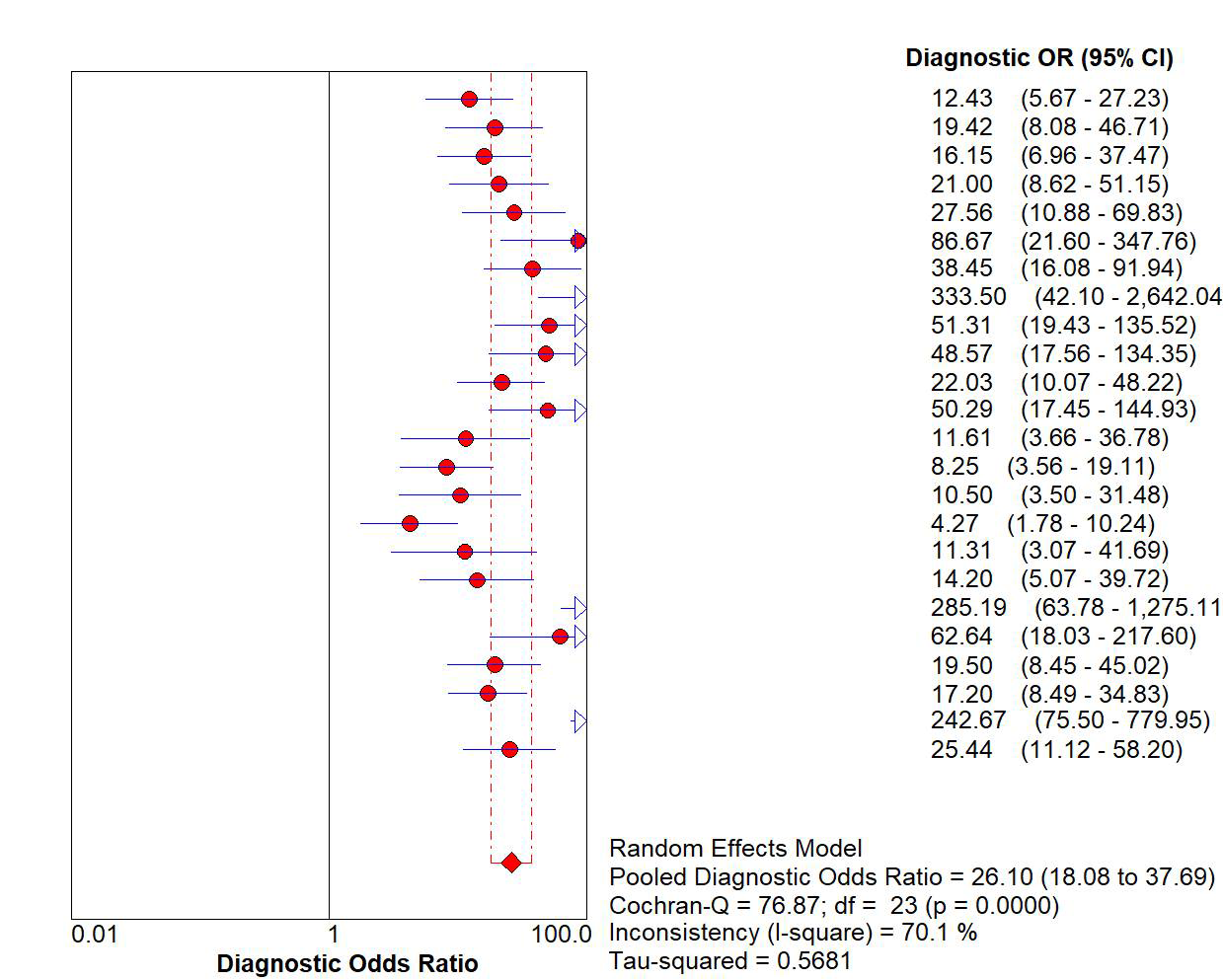

Supplement: Supplementary file 1 [file Image_1.TIF]
